# Dubin-Johnson Syndrome: A Case Report

**DOI:** 10.7759/cureus.36115

**Published:** 2023-03-14

**Authors:** Abdul Hannan Siddiqui, Muhammad R Alsabe, Zuha Tehseen, Modather I Hatamleh, Sanzida Taslim, Ameer Abdelrahman, Faraz Saleem

**Affiliations:** 1 Internal Medicine, University Hospital Derby and Burton, Derby, GBR; 2 Medicine, Ross University School of Medicine, Queens, USA; 3 Internal Medicine, California Institute of Behavioral Neurosciences and Psychology, California, USA; 4 Internal Medicine, King Abdullah bin Abdulaziz University Hospital, Amman, JOR; 5 Internal Medicine, Ross University School of Medicine, Far Rockaway, USA; 6 Medicine, Inova Alexandria Hospital, Virginia, USA

**Keywords:** #hyperbilirubinemia, #conjugated hyperbilirubinemia, #liver, #liver abnormalities, liver histopathology, djs, dubin jhonson syndrome

## Abstract

Dubin-Johnson syndrome (DJS) is a rare autosomal recessive genetic disease caused by mutations in the bilirubin transporter MRP2. It is characterized by recurrent episodes of jaundice and conjugated hyperbilirubinemia. Numerous instances of hyperbilirubinemia disorders resembling Dubin-Johnson syndrome have been documented, but they differ in the clinical presentation, amount of conjugated bilirubin present, and their reaction to therapy. Most people with this syndrome do not have any symptoms, so their cases are often misdiagnosed and not properly taken care of. Here, we present a case of a teenage male patient who complained of recurring jaundice and abdominal pain. Further examination and testing revealed that the patient had been jaundiced since birth and had a family history of the condition. Conservative management was implemented, and follow-up demonstrated a positive prognosis. This case is a rare example of Dubin-Johnson syndrome, although patients with the condition generally have a normal life expectancy and only require conservative management.

## Introduction

Dubin-Johnson syndrome (DJS) is an autosomal recessive inherited disorder that causes high levels of conjugated bilirubin and changes the way the body processes coproporphyrins, leading to increased urinary excretion of coproporphyrin I relative to coproporphyrin III. DJS is caused by a mutation in the ATP Binding Cassette Subfamily C Member 2 (ABCC2) gene, which codes for the multi-drug resistance protein 2 (MRP2) bilirubin transporter and is primarily present in the canalicular membrane of hepatocytes [1,2]. In 1954, Dubin and Johnson documented 12 cases of persistent idiopathic jaundice with an unidentified pigment in the liver as a new clinical and pathological entity [3].

Most individuals with Dubin-Johnson syndrome are asymptomatic, and its main symptom is mild icterus. Patients may experience minor problems such as persistent stomach aches and weakness. Icterus may occasionally be so mild that it is only noticeable during concurrent diseases, pregnancy, or oral contraceptive use [4]. The gold standard test for diagnosis is a liver biopsy. On gross inspection, the liver appears black in Dubin-Johnson syndrome, despite the liver architecture being normal. However, centrilobular hepatocytes have an overabundance of a dark, granular pigment. Under electron microscopy, the pigment is seen to be inside the lysosomes. Based on the pigment's histochemical staining and physicochemical characteristics, it is assumed to be related to melanin [1,3].

The prevalence of DJS is estimated to be less than one case per 100,000 individuals globally, but it is more common among Iranian Jews. Although it is considered a rare disorder worldwide, the incidence of the condition among Sephardic Jews is approximately 1 in 3000, according to reports [1,2]. Many individuals with DJS are typically young adults and do not display any symptoms. Both sexes have equal chances of having Dubin-Johnson syndrome; however, in females, the disease may not show any apparent signs and may only be detected due to elevated bilirubin levels or noticeable jaundice after beginning oral contraceptives or becoming pregnant. During this time, jaundice can result in the identification of the diagnosis [3]. Dubin-Johnson syndrome is a benign condition that does not have long-term consequences and typically does not require medical treatment. However, it is important to diagnose DJS to rule out other hepatobiliary disorders that may cause liver damage, some of which may be treatable if identified [3].

## Case presentation

A 17-year-old Asian male patient presented to the outpatient department of a tertiary care hospital with recurrent episodes of jaundice in the past few weeks. The jaundice was mild and sporadic, and the patient also complained of generalized abdominal pain, with most of the discomfort felt in the upper right quadrant of his abdomen. He denied having a fever, nausea, vomiting, or any oral intake of medicine. Physical examination revealed a pale yellow body colour, while his urine was dark yellow and his stool was brown. The past medical history of the patient was unremarkable for any illnesses, and he was never admitted to the hospital. However, the patient did report intermittent and occasional abdominal pain and yellowness of the skin two years ago, for which he underwent general laboratory investigations after consulting his physician. His labs were unremarkable except for the high conjugated bilirubin level. As the patient's condition resolved on its own without consultation with his physician, he did not follow up on his laboratory results. The patient had a family history of the same condition, and previous tests indicated elevated levels of conjugated bilirubin. All vital signs were normal, and laboratory investigations showed an elevated total bilirubin level of 61 µmol/L and direct conjugated bilirubin of 40 µmol/L. Aspartate aminotransferase was 28 U/L, alkaline phosphatase was 160 U/L, and the reticulocyte count was 1.5%. Serological tests for hepatitis A and B were negative (Table 1).

**Table 1:** Laboratory investigations of the patient.

Sr. No.	Laboratory investigation	Values	Normal Values
1	Total serum bilirubin	61 µmol/L	1.70–20.00 µmol/L
2	Direct conjugated bilirubin	40 µmol/L	5.1 µmol/L or less
3	Urine bilirubin	1.4 mg/dl	0.3–1.0 mg/dl
4	Serum aspartate aminotransferase	28 IU/L	38 IU/L or less
5	Serum alkaline phosphatase	160 IU/L	500 IU/L or less
6	Reticulocyte count	1.5%	0.5–2.5%
7	Coproporphyrin I	49	30 (µg/L) or less
8	Coproporphyrin III	153	150 µg/24 h or less

**Figure 1:**
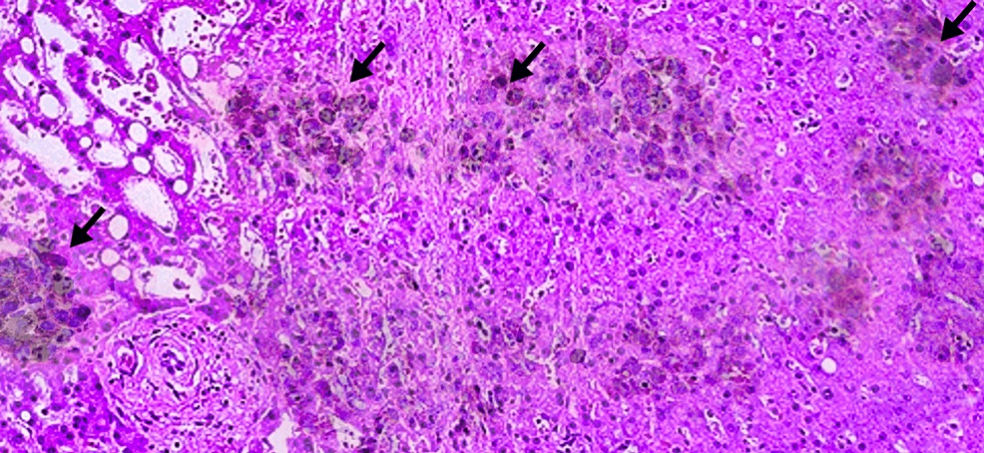
Liver histology showing significant intracellular pigments in the centrilobular hepatocytes (H&E stain).

The ultrasound scan showed a healthy liver without any signs of biliary obstruction or organ enlargement. The patient's other liver function tests were also found to be within the normal range. Based on his past medical history, laboratory tests, and clinical evaluation, a diagnosis of Dubin-Johnson syndrome was suspected. A liver biopsy was conducted, which revealed melanin pigment inside the liver parenchyma, leading to the liver turning black (Figure 1).

After a thorough evaluation of the patient's history, clinical symptoms, physical examination, laboratory tests, ultrasound scans, and liver biopsy results, a definitive diagnosis of Dubin-Johnson syndrome was confirmed. Unfortunately, there is currently no known cure for this condition. Therefore, the patient was prescribed symptomatic treatment, which included a drug therapy regimen of ursodeoxycholic acid (UDCA) 300 mg and rifampicin 600 mg in divided dosages (two times a day) to manage the hyperbilirubinemia. To alleviate the patient's abdominal pain, Ibuprofen 400 mg (single dose) was also prescribed. Follow-up liver function tests after a month of treatment showed a decrease in conjugated bilirubin levels to 29 µmol/L. The treatment was continued for a total of three months, and the medications were tapered off accordingly. The patient was instructed to return to the specialist for long-term management and follow-up. Furthermore, the patient received counseling on genetic and lifestyle modifications, including dietary modification, ensuring appropriate hydration, and avoiding triggers such as stress and alcohol consumption, to manage his condition effectively.

## Discussion

Dubin-Johnson syndrome is a relatively rare hereditary disorder that is inherited in an autosomal recessive manner. The prevalence of DJS is estimated to be less than one case per 100,000 individuals globally, but it is more common among Iranian Jews. Although it is considered a rare disorder worldwide, the incidence of the condition among Sephardic Jews is approximately 1 in 3000, according to reports. The disorder is characterized by hyperbilirubinemia, primarily an excess of the conjugated fraction, and a change in the way coproporphyrins are metabolized, causing individuals with DJS to excrete more coproporphyrin I than III in their urine. The underlying cause of DJS is a mutation in multidrug resistance protein-2 (MRP2), a bilirubin transporter responsible for transporting conjugated bilirubin into the bile duct. The ABCC2 gene on chromosome 10q24 encodes MRP2, and a mutation in this gene can impair the protein's function, leading to DJS. Recent studies have confirmed that the ABCC2 gene mutation is responsible for the loss of MRP2 function, which causes DJS in affected patients. [1-3,5].

DJS typically affects young adults who are asymptomatic, although hyperbilirubinemia is often detected incidentally during routine or unrelated medical testing. Mild symptoms such as icterus, weakness, and/or upper abdominal pain may occur in rare cases. Unlike other conditions that cause hyperbilirubinemia, such as liver disease, DJS does not typically cause pruritus, as serum total bile acid levels remain normal. In some cases, women with the condition may not exhibit any symptoms, but hyperbilirubinemia or obvious jaundice may develop when they start using oral contraceptives or become pregnant [6].

In this case report, a patient presented with recurring jaundice and upper right quadrant abdominal pain, which prompted a detailed examination and history to diagnose the underlying issue. A liver biopsy was performed to confirm the diagnosis, which revealed dark brown pigment in the hepatocytes. The liver in DJS appears grey to black due to the widespread buildup of coarsely granular pigment, which is brown in colour in the cytoplasm of hepatocytes [7]. In the biopsy, differential diagnoses such as an iron overload condition and lipofuscin were ruled out.

It is important to note that the treatment of DJS should be individualized based on the patient's symptoms and response to therapy. Close monitoring of liver function tests and bilirubin levels is essential to adjust the medication doses and assess the treatment's effectiveness. Phenobarbital and ursodeoxycholic acid are medications that can help treat jaundice in patients with Dubin-Johnson syndrome (DJS) [3]. Phenobarbital increases bilirubin excretion, and the dose varies based on symptom severity and patient response. A starting dose of 15 to 30 mg/kg/day is typically given and then gradually increased until the desired effect is achieved. Ursodeoxycholic acid improves bile flow and has a hepatoprotective effect, and the recommended dose for DJS is 15 mg/kg/day in divided doses. Rifampicin is also one of the drugs that have shown improvement in hyperbilirubinemia in patients with DJS. Typically, the recommended dose of rifampicin for the treatment of DJS is 10 mg/kg/day, or 600 mg/day, administered orally and in divided doses. However, rifampicin should be used with caution in DJS because it can cause liver toxicity and interact with other medications [8,9].

The clinical manifestations of DJS are often minimal and have a favourable prognosis. The symptoms of DJS with an origin in infancy may last into childhood, but they do not often interfere with growth and development. Long-term pigment deposition in hepatocytes, however, causes the rupture of bile ducts in the liver, leading to hepatocyte degeneration and necrosis, the proliferation of fibrous tissue, pseudo-lobule development, and a variety of clinical alterations. Thus, jaundice should be actively addressed in pediatric patients with severe jaundice and recurrent DJS in order to limit the accumulation of pigments in the hepatocytes and prevent the exacerbation of harm to the hepatocytes. Phenobarbital, ursodeoxycholic acid, and rifampicin should be evaluated as pharmacological therapies for reducing jaundice, protecting the liver, and lowering enzyme levels, thereby decreasing clinical symptoms and the risk of hepatocyte injury [10].

In one of the studies, the long-term follow-up of the pediatric population showed delayed developmental milestones. In June 2020, follow-up evaluations showed that case 1's cholestasis had gradually improved, and they had normal growth and development but still had mild jaundice of the skin. Case 2's cholestasis had also improved, and jaundice had almost disappeared, but growth and development were delayed. Case 3's cholestasis had improved, and her jaundice had mostly disappeared, but her growth and development were also delayed, and she had below-average weight and height measurements [11].

Four cases of DJS, two diagnosed during the neonatal period and two diagnosed during adolescence, were followed for 5-20 years. Mutational analysis in the MRP2/ABCC2 gene was performed in all four cases. A biphasic pattern of jaundice attack was observed in one patient who was followed for 20 years. Thus, long-term follow-up of a neonatal-onset case is mandated because DJS may have a biphasic pattern of jaundice attack, with a second attack occurring after adolescence [12].

DJS is a non-threatening condition that does not advance to fibrosis or cirrhosis and does not necessitate any medical intervention. The significance of identifying DJS is to rule out the likelihood of other hepatobiliary disorders that could cause damage to the liver and identify those that might be treatable.

## Conclusions

Dubin-Johnson syndrome is a rare genetic condition that poses substantial diagnostic challenges for physicians. However, it can be detected with a complete history, particularly the family history, clinical symptoms, and laboratory studies. Genetic testing must be performed for a specific diagnosis. The diagnosis is confirmed by a liver biopsy, and a multimodal care strategy is required, including counselling for lifetime dietary modification, ensuring appropriate hydration, and avoiding triggers such as stress, alcohol, and pregnancy. Although there is no known cure for DJS, some medications, such as phenobarbital, UDCA, and rifampicin, are common medications prescribed for the treatment of DJS. Regular follow-up is important to monitor the patient's condition and prevent the progression of the disease.
